# Nomenclature for kidney function and disease: executive summary and glossary from a Kidney Disease: Improving Global Outcomes (KDIGO) consensus conference

**DOI:** 10.1007/s10157-020-01946-0

**Published:** 2020-08-19

**Authors:** Andrew S. Levey, Kai-Uwe Eckardt, Nijsje M. Dorman, Stacy L. Christiansen, Michael Cheung, Michel Jadoul, Wolfgang C. Winkelmayer

**Affiliations:** 1 Division of Nephrology, Tufts Medical Center, 800 Washington Street, Box 391, Boston, MA 02111 USA; 2 Department of Nephrology and Medical Intensive Care, Charité-Universitätsmedizin Berlin, Augustenburger Platz 1, 13353 Berlin, Germany; 3 American Journal of Kidney Diseases, Philadelphia, USA; 4 Journal of the American Medical Association, Chicago, USA; 5 Kidney Disease: Improving Global Outcomes (KDIGO), Brussels, Belgium; 6 Cliniques Universitaires Saint Luc, Université Catholique de Louvain, Brussels, Belgium; 7 Selzman Institute for Kidney Health, Section of Nephrology, Department of Medicine, Baylor College of Medicine, Houston, TX USA

A primary obligation of medical journals is the responsible, professional, and expeditious delivery of knowledge from researchers and practitioners to the wider community [1]. The task of journal editors, therefore, rests not merely in selecting what to publish, but in large measure judging how it can best be communicated. The challenge of improving descriptions of kidney function and disease in medical publishing was the impetus for a Kidney Disease: Improving Global Outcomes (KDIGO) Consensus Conference held in June 2019. The conference goals included standardizing and refining kidney-related nomenclature used in English-language scientific articles and developing a glossary that can be used by journals [2].

The rationale for the conference was that the worldwide burden of kidney disease is rising, but public awareness remains limited, underscoring the need for effective communication by stakeholders in the kidney health community [3–6]. Despite this need, the nomenclature for describing kidney function and disease lacks uniformity and clarity. Two decades ago, a survey of hundreds of published articles and meeting abstracts reported a broad array of overlapping, confusing terms for chronic kidney disease (CKD) and advocated adoption of unambiguous terminology [7]. Nevertheless, terms flagged by that analysis as problematic, such as “chronic renal failure” and “pre-dialysis,” still appear in current-day publications. A coherent, shared nomenclature could improve communication at all levels, to not only foster better appreciation of the burden of disease but also aid understanding of how patients feel about their disease, allow more effective communication between kidney disease specialists and other clinicians, advance more straightforward comparison and integration of datasets, enable better recognition of gaps in knowledge for future research, and facilitate more comprehensive public health policies for acute and chronic kidney disease.

Developing consistent, patient-centered, and precise descriptions of kidney function and disease in the scientific literature is an important objective to align communication in clinical practice, research, and public health. Although some terms have been in use for decades, the increased exchange of information among stakeholders makes it timely to revisit nomenclature to ensure consistency. The goal is to facilitate communication within and across disciplines and between practitioners and patients, with the ultimate hope of improving outcomes through consistency and precision.

Attendees at the conference included editors of kidney subspecialty journals, kidney subspecialty editors at general medical journals and journals from other subspecialties, experienced authors of clinical kidney health research, and patients. Guiding principles of the conference were that the revised nomenclature should be patient-centered, precise, and consistent with nomenclature used in the KDIGO guidelines. The discussion focused on general description of acute and chronic kidney disease and kidney measures, rather than specific kidney diseases and particular measures of function and structure. Classifications of causes of kidney disease and procedures, performance measures, and outcome metrics for dialysis and transplantation were considered beyond the scope of discussion.

As described in detail in the conference report [8], the meeting attendees reached general consensus on the following recommendations: (1) to use “kidney” rather than “renal” or “nephro-” when referring to kidney disease and kidney function; (2) to use “kidney failure” with appropriate descriptions of presence or absence of symptoms, signs, and treatment rather than “end-stage kidney disease”; (3) to use the KDIGO definition and classification of acute kidney diseases and disorders (AKD) and acute kidney injury (AKI) rather than alternative descriptions to define and classify the severity of these; (4) to use the KDIGO definition and classification of CKD rather than alternative descriptions to define and classify it; and (5) to use specific kidney measures, such as albuminuria or decreased glomerular filtration rate, rather than “abnormal” or “reduced” kidney function to describe alterations in kidney structure and function (Table 1). Accordingly, the proposed glossary contains five corresponding sections, and comprises specific items for which there was general agreement among the conference participants (https://kdigo.org/conferences/nomenclature/; Table 2) [8]. For each section, the glossary includes preferred terms, abbreviations, descriptions, and terms to avoid, with the acknowledgment that journals may choose which of the recommendations to implement, and that journal style will dictate when and how to abbreviate terms to be consistent with nomenclature for other diseases.

**Table 1:** Key takeaways from the conference

Use the term “kidney” rather than “renal” to describe kidney function and kidney disease. In English, the terms renal and kidney are still used interchangeably, resulting in different acronyms describing the same condition or status (e.g., ESRD/ESKD and RRT/KRT). It is more likely that patients and the public would understand the terms incorporating the more familiar noun “kidney,” rather than the less familiar adjective “renal,” which is derived from Latin and is labeled as technical in some dictionaries. Although writing guides may generally favor using an appropriate adjective over a noun as a modifier, there are high-profile precedents for the use of kidney as a modifier, such as AKI, CKD, and NIDDK (National Institute of Diabetes and Digestive and Kidney Diseases)
Avoid the term “end-stage.” Although rooted in US law, the term is not patient sensitive, may connote a stigma, and may discourage advocacy. In the US, ESRD (ESKD) is a synonym for receipt of KRT. However, KRT is a treatment rather than a disease. The term “kidney failure,” which is defined as GFR < 15 ml/min per 1.73 m^2^ or treatment by dialysis, is as comprehensive as “ESRD/ESKD,” without suffering from its limitations
Improve characterization of the full spectrum of kidney failure. Although all patients with kidney failure have GFR < 15 ml/min per 1.73 m^2^ or are undergoing treatment by dialysis, the severity of symptoms varies greatly. We lack terms to describe the severity of symptoms and signs, and yet they are indications for initiating KRT. There are also no common patient-reported outcome measures to describe severity. The term “kidney failure” in a chronic setting is defined as > 3 months, whereas in an acute setting (i.e., AKI stage 3), it is reserved for a duration of ≤ 3 months. Kidney failure could be further classified according to patient-reported outcomes (symptoms)
Use more-descriptive terms for treatments for kidney failure. Many patients with kidney failure do not undergo KRT. The terms “treated” vs. “untreated” have been used, but this is not consistent with the idea that supportive care is indeed treatment. Furthermore, in some cases, patients choose supportive care rather than KRT; in other cases, they do not have a choice because of lack of insurance or lack of availability. Finally, some patients may not be under the care of a physician at all
Avoid the use of “chronic kidney disease (CKD)” as a synonym for “GFR < 60 ml/min per 1.73 m^2^.” CKD includes markers of kidney damage or GFR < 60 ml/min per 1.73 m^2^ for > 3 months, so ascertainment of GFR without assessment for markers of kidney damage is insufficient for classification of CKD status when GFR > 60 ml/min per 1.73 m^2^. If chronicity is not documented, it can be inferred on the basis of corroborative clinical data or presumed in the absence of clinical data to the contrary
Avoid the use of “acute kidney injury (AKI)” as a synonym for “acute kidney diseases and disorders (AKD).” AKD refers to kidney diseases and disorders with a duration of ≤ 3 months, whereas AKI refers to kidney diseases and disorders with onset within 1 week
Use “CKD GFR and albuminuria categories” and “AKI stages” to describe disease severity, rather than employing ill-defined terms, such as “mild,” “moderate,” “severe,” and “advanced”
Use the terms “GFR categories” and “albuminuria categories” rather than “CKD stages” when describing the level of GFR and albuminuria in populations either without CKD or without ascertainment of both GFR and albuminuria
Use the term “risk categories” to describe combinations of the G (GFR) and A (albuminuria) categories from the KDIGO heat map (see Supplementary Figure S1)
Use specific terms, such as “GFR,” “tubular secretion,” “tubular reabsorption,” “albuminuria,” and “proteinuria,” rather than general terms, such as “abnormal” or “reduced” kidney function, damage, or injury, when possible. Because kidney function comprises several functional categories, including excretory, endocrine, and metabolic functions, it should be described as specifically as possible. GFR is closely linked with the excretory function, but it should not be used as a synonym, because tubular reabsorption and excretion also contribute to excretory function
When referring to “decreased or decreasing GFR,” avoid the use of different, poorly defined terms, such as “impaired kidney function,” “renal insufficiency,” “renal dysfunction,” “renal impairment,” “worsening kidney function,” and “kidney function decline”
When referring to GFR, use descriptive abbreviations (mGFR for measured GFR and eGFR for estimated GFR, with specific notation based on the endogenous filtration markers used (e.g., eGFR_cr_, eGFR_cys_, and eGFR_cr-cys_). Additional detail can be given in the methods. For mGFR, the methods should describe the exogenous filtration marker (e.g., inulin, iothalamate, iohexol) and clearance method (urinary clearance, plasma clearance). For eGFR, the methods should describe the estimating equation used (CKD-EPI; MDRD Study)
Avoid referring to “albuminuria” or “proteinuria” as “decreased kidney function.” Albuminuria and proteinuria are markers of kidney damage, rather than measures of kidney function
When referring to albuminuria or proteinuria, avoid the terms “microalbuminuria” and “macroalbuminuria/clinical proteinuria.” Use the terms “moderately increased” or “severely increased” instead
When referring to albuminuria and proteinuria, use descriptive abbreviations, such as “urine albumin or protein excretion rates (AER and PER)” and “urine albumin–creatinine or protein–creatinine ratios (ACR and PCR)”

*ACR* albumin–creatinine ratio, *AER* albumin excretion rate, *AKD* acute kidney diseases and disorders, *AKI* acute kidney injury, *CKD* chronic kidney disease, *CKD-EPI* CKD Epidemiology Collaboration, *eGFR* estimated glomerular filtration rate, *eGFR*_*cr*_ estimated glomerular filtration rate derived from creatinine, *eGFR*_*cr-cys*_ estimated glomerular filtration rate derived from creatinine and cystatin C, *eGFR*_*cys*_ estimated glomerular filtration rate derived from cystatin C, *ESKD* end-stage kidney disease, *ESRD* end-stage renal disease, *GFR* glomerular filtration rate, *KDIGO* Kidney Disease: Improving Global Outcomes, *KRT* kidney replacement therapy, *MDRD* modification of diet in renal disease, *mGFR* measured glomerular filtration rate, *NIDDK* National Institute of Diabetes and Digestive and Kidney Diseases, *PCR* protein-creatinine ratio, *PER* protein excretion rate, *RRT* renal replacement therapy, *US* United States

**Table 2:** KDIGO Kidney Function and Disease Glossary: suggested terms to describe kidney function and kidney disease, and criteria and measures defining them

**Preferred term**	**Suggested abbreviations**^**a**^	**Rationale/explanation**	**Terms to avoid**
**Part 1. Kidney function and disease**		The term “kidney” should be used preferentially when describing kidney disease and kidney function, with exceptions	“Renal,” the prefix “nephro-” (except in the setting of specific functions, diseases, or syndromes; see below)
**Kidney disease**		Reflects the entirety of acute kidney diseases and disorders and chronic kidney disease	Renal disease, nephropathy (except in the setting of specific diseases, e.g., membranous nephropathy)
**Kidney function**		Reflects the entirety of different and complex physiological functions of the kidney; should not be equated with glomerular filtration rate (GFR) only	Renal function (except when describing specific functions, e.g., renal acidification, renal concentrating mechanism)
Normal kidney function		General term applicable to various aspects of kidney function that should be specified	
Abnormal kidney function		General term applicable to various aspects of kidney function that should be specified	Renal/kidney impairment, insufficiency, dysfunction; azotemia
Residual kidney function	RKF	Kidney function in people with kidney failure receiving KRT; further specification is required, e.g., urine flow rate, solute clearance. Although it is usually used in the setting of dialysis, this term could be used to refer to native kidney function in kidney transplant recipients	Residual renal function (RRF)
**Kidney structure**		Reflects the entirety of different and complex structures of the kidney, ascertained by imaging and markers of injury and damage	Renal structure (except when describing specific structures within the kidney, such as artery, vein, capsule, parenchyma, cortex, medulla, glomeruli, tubules, interstitium, cysts, tumors)
Normal kidney structure		General term applicable to various aspects of kidney structure that should be specified	
Abnormal kidney structure		General term applicable to various aspects of kidney structure that should be specified	
**Causes of kidney disease**		Cause of AKI, AKD, and CKD should be indicated whenever possible. Cause may be known, presumed, or unknown. Method for ascertainment and attribution of cause should be specified	Cause should not be inferred only from presence of comorbid condition (such as diabetes)
**Part 2. Kidney failure**		GFR < 15 ml/min per 1.73 m^2^ or treatment by dialysis; further specification is required; see below	Renal failure (RF); end-stage renal disease (ESRD); end-stage kidney disease (ESKD); renal disease; nephropathy; renal/kidney impairment, insufficiency, dysfunction; azotemia
**Duration**		Specification preferred	
Acute kidney injury stage 3^b^	AKI stage 3	Disease duration ≤ 3 months	Acute renal failure; renal disease; nephropathy; renal/kidney impairment, insufficiency, dysfunction; azotemia; uremia
Kidney failure	KF	Disease duration > 3 months	Chronic renal failure; chronic renal disease; chronic nephropathy; chronic renal/kidney impairment, insufficiency, dysfunction; azotemia; uremia; irreversible kidney failure
**Symptoms and signs**		Specification preferred (with, without, or unknown symptoms and signs); with symptoms and signs would be synonymous with uremia	
Uremia/uremic syndrome		A syndrome consisting of symptoms and signs associated with kidney failure (does not indicate a causal role for urea)	
**Treatment**		Specification required	
Kidney replacement therapy^c^	KRT	Further specification is required; includes dialysis and transplantation	Renal replacement therapy (RRT)
Dialysis	AKI stage 3D	AKI stage 3 treated by dialysis	AKI-D, dialysis-dependent AKI
	CKD G5D	CKD G5 treated by dialysis	ESKD, ESKF, ESRD, ESRF, dialysis-dependent CKD
Duration		Long-term vs. short-term: long-term refers to dialysis for CKD, and may also be referred to as maintenance dialysis; short-term refers to dialysis for AKD	Chronic dialysis, acute dialysis (the terms acute and chronic refer to duration of kidney disease rather than duration of dialysis treatment)
Modality and frequency		Modalities - Hemodialysis (HD) - Hemofiltration (HF) - Hemodiafiltration (HDF) - Peritoneal dialysis (PD, ambulatory or automated) Frequency - Continuous - Intermittent (short or prolonged)	
Kidney transplantation	CKD G1T–G5T	CKD G1–G5 after transplantation	ESKD, ESKF, ESRD, ESRF
Donor source		Specify living donor kidney transplant/transplantation (LDKT) or deceased donor kidney transplant/transplantation (DDKT)	
Kidney failure with replacement therapy	KFRT	CKD G5 treated by dialysis or CKD G1–G5 after transplantation; for epidemiologic studies, both should be included	ESKD, ESKF, ESRD, ESRF
Kidney failure without replacement therapy	CKD G5 without KRT	Further specification is preferred: specify whether KRT is not chosen vs. not available	ESKD, ESKF, ESRD, ESRF, untreated kidney failure
With comprehensive conservative care		Further specification is preferred; definition is evolving	
Without comprehensive conservative care		Further specification is preferred: specify whether comprehensive conservative care is not chosen vs. not available	
**Part 3. Acute Kidney Diseases and Disorders (AKD) and Acute Kidney Injury (AKI)**		Disease duration ≤ 3 months; conceptually different from initial recognition of CKD	Acute renal failure (ARF); acute renal insufficiency (ARI)
**Acute kidney diseases**	AKD^c^	KDIGO definition: AKI, or GFR < 60 ml/min per 1.73 m^2^, or markers of kidney damage for ≤ 3 months, or decrease in GFR by ≥ 35% or increase in SCr by > 50% for ≤ 3 months	ARF, ARI
**Acute kidney injury**	AKI	KDIGO definition (AKI is a subcategory of AKD): oliguria for > 6 h, rise in SCr by > 0.3 mg/dl in 2 days or by > 50% in 1 week	ARF, ARI
**AKI classification**		KDIGO classification by cause and stage preferred rather than stage alone; e.g., a patient with AKI stage 3 due to ATN; classification applies to all AKI stages	Previous classifications, including RIFLE and AKIN (the KDIGO classification harmonized these prior definitions)
**AKI stages**		KDIGO definition (applicable only to people with AKI)	
	AKI stage 1	Serum creatinine and/or urine output criteria	
	AKI stage 2	Serum creatinine and/or urine output criteria	
	AKI stage 3	Serum creatinine and/or urine output criteria	
**Part 4. Chronic Kidney Disease (CKD)**		Disease duration > 3 months	Chronic renal failure (CRF); ESRD; renal/kidney impairment, insufficiency, dysfunction
**CKD**		KDIGO definition: GFR < 60 ml/min per 1.73 m^2^ or markers of kidney damage for > 3 months	CRF; ESRD; renal/kidney impairment, insufficiency, dysfunction
**CKD classification**		KDIGO CGA classification by cause, GFR category (G1–G5), and albuminuria category (A1–A3); see below for definitions of G and A categories. For example, a patient with CKD G1, A3 due to diabetes, or a cohort with CKD G4–G5, A1–A3 of any cause. Note that CKD classification is only applicable to people with CKD, so a patient could not be classified as “CKD G2, A1” if there was no other evidence of kidney damage	Mild, moderate, severe, early, advanced CKD; CKD stage 1–5 (complete description preferred rather than G category alone)
CKD without KRT	CKD without KRT	CKD G1–G5, A1–A3 of any cause, not receiving dialysis or transplantation	ND-CKD (non-dialysis CKD), NDD-CKD (non–dialysis-dependent CKD), predialysis CKD, pre-ESRD CKD
**CKD risk categories**		KDIGO definitions (colors refer to heat map in Supplementary Figure S1) unless otherwise defined; risk depends on the outcome being considered	Mild, moderate, severe, early, advanced CKD
CKD risk category—low	Low risk	Refers to G1A1, G2A1 (green)	
CKD risk category—moderately high	Moderate risk	Refers to G1A2, G2A2, G3aA1 (yellow)	
CKD risk category—high	High risk	Refers to G1A3, G2A3, G3aA2, G3bA1 (orange)	
CKD risk category—very high	Very high risk	Refers to G3aA3, G3bA2, G3bA3, G4A1, G4A2, G4A3, G5A1, G5A2, G5A3 (red)	
**CKD progression**		Refers to worsening GFR or albuminuria. Other biomarkers not included. There is not yet consensus on use of specific terms to describe the timing (e.g., early, late) or rate (fast, slow) of progression. Use of specific terms should be defined in methods Further specification may be required: GFR decline may occur during therapy for other conditions, which may not be considered as CKD progression	
**CKD remission**		Refers to improving GFR or albuminuria. Criteria depend on disease. Use of specific terms should be defined in methods	
**Part 5. Kidney Measures**		Applies to people with or without kidney disease; consider measurement issues (methods) and variability (multiple measures may improve classification)	
**Glomerular filtration rate and clearance**		GFR and creatinine clearance are not synonymous	
Glomerular filtration rate	GFR	Units must be specified (ml/min per 1.73 m^2^ or ml/min)	
Measured glomerular filtration rate	mGFR	Clearance methods and exogenous filtration markers should be noted separately in methods	
Estimated glomerular filtration rate	eGFR	Estimating equations (e.g., CKD-EPI and MDRD Study) and filtration markers (e.g., creatinine and cystatin C) should be noted separately in methods	
Estimated glomerular filtration rate; marker	eGFR_cr_	eGFR using creatinine	
	eGFR_cys_	eGFR using cystatin C	
	eGFR_cr-cys_	eGFR using creatinine and cystatin C	
Clearance	Cl	Solute must be specified; units must be specified (ml/min per 1.73 m^2^ or ml/min)	
Measured clearance	mCl	Clearance methods and markers should be noted separately in methods	
Measured clearance; marker	mCl_UN_	mCl using urea nitrogen	
	mCl_cr_	mCl using creatinine	
	mCl_UN-cr_	mCl using urea nitrogen and creatinine	
Estimated clearance	eCl	Estimating equations (e.g., Cockcroft-Gault) and markers should be noted separately in methods	
Estimated clearance; marker	eCl_cr_	eCl using creatinine	
**GFR categories**		For use in describing GFR level irrespective of the presence or absence of kidney disease; GFR units are ml/min per 1.73 m^2^ for these categories; multiple categories can be collapsed (e.g., G3–G5)	
Normal to increased GFR	G1	GFR ≥ 90 ml/min per 1.73 m^2^	
Mildly reduced GFR	G2	GFR 60–89 ml/min per 1.73 m^2^	
Moderately reduced GFR	G3a	GFR 45–59 ml/min per 1.73 m^2^	
	G3b	GFR 30–44 ml/min per 1.73 m^2^	
Severely reduced GFR	G4	GFR 15–29 ml/min per 1.73 m^2^	
Kidney failure	G5	GFR < 15 ml/min per 1.73 m^2^ or treated by dialysis	
Hyperfiltration		The concept of hyperfiltration is generally accepted but not consistently defined. If this term is used as an exposure, outcome, or covariate, the GFR threshold must be defined (e.g., > 120 ml/min per 1.73 m^2^)	Renal hyperfiltration
GFR reserve		The concept of GFR reserve is generally accepted as the difference between stimulated and basal GFR	Renal function reserve
**Albuminuria and proteinuria**		Specify measurement conditions (spot vs. timed samples; quantitative vs. dipstick); differentiate non-albumin proteins as clinically indicated	
**Albuminuria**			Microalbuminuria, macroalbuminuria
Urinary albumin concentration			
Urinary albumin excretion rate	AER	Requires timed urine collection; interval for urine collection should be noted separately in methods; unit of time may vary (hour or day)	
Urinary albumin-creatinine ratio	ACR	From timed urine collection or spot urine collection; interval for timed urine collection, or time of day for spot urine collection, should be noted separately in methods	
**Proteinuria**			Clinical proteinuria, overt proteinuria
Urinary protein concentration			
Urinary protein excretion rate	PER	Requires timed urine collection; interval for urine collection should be noted separately in methods; unit of time may vary (hour or day)	
Urinary protein-creatinine ratio	PCR	From timed urine collection or spot urine collection; interval for timed urine collection, or time of day for spot urine collection, should be noted separately in methods	
**Albuminuria and proteinuria categories**		For use in describing albuminuria or proteinuria level irrespective of the presence or absence of kidney disease	
Normal		AER < 10 mg/day; ACR < 10 mg/g (< 1 mg/mmol)	Normoalbuminuria
Mildly increased (mild)		AER 10–29 mg/day; ACR 10–29 mg/g (1.0–2.9 mg/mmol)	
Normal to mildly increased (normal to mild)	A1	AER < 30 mg/day; ACR < 30 mg/g (< 3 mg/mmol) PER < 150 mg/day; PCR < 150 mg/g (< 15 mg/mmol)	
Moderately increased (moderate)	A2	AER 30–300 mg/day; ACR 30–300 mg/g (3–30 mg/mmol) PER 150–500 mg/day; PCR 150–500 mg/g (15–50 mg/mmol)	Microalbuminuria
Severely increased (severe)	A3	AER > 300 mg/day; ACR > 300 mg/g (> 30 mg/mmol) PER > 500 mg/day; PCR > 500 mg/g (> 50 mg/mmol)	Macroalbuminuria, clinical proteinuria, overt proteinuria
Nephrotic-range/syndrome^d^		AER > 2200 mg/day; ACR > 2200 mg/g (> 220 mg/mmol) PER > 3500 mg/day; PCR > 3500 mg/g (> 350 mg/mmol) Specify with or without nephrotic syndrome, as noted by the presence of hypoalbuminemia (with edema and hyperlipidemia in most cases)	
**Tubular function**			
Tubular secretion	TS	Further specification is required to distinguish rate, clearance, or fraction (compared to filtered load)	
Tubular reabsorption	TR	Further specification is required to distinguish rate, clearance, or fraction (compared to filtered load)	
Fractional excretion, marker	FE_Na_	FE of sodium	
Fractional reabsorption, marker	FR_Na_	FR of sodium	

*ACR* albumin–creatinine ratio, *AER* albumin excretion rate, *AKD* acute kidney diseases and disorders, *AKI* acute kidney injury, *AKIN* Acute Kidney Injury Network, *ARF* acute renal failure, *ARI* acute renal insufficiency, *ATN* acute tubular necrosis, *CGA* cause *GFR* category and albuminuria category, *CKD* chronic kidney disease, *CKD-EPI* CKD Epidemiology Collaboration, *DDKT* deceased donor kidney transplant/transplantation, *eGFR* estimated glomerular filtration rate, *ESKD* end-stage kidney disease, *ESKF* end-stage kidney failure, *ESRD* end-stage renal disease, *ESRF* end-stage renal failure, *FE*_*Na*_ fractional excretion sodium, *FR*_*Na*_ fractional reabsorption sodium, *GFR* glomerular filtration rate, *HD* hemodialysis, *HDF* hemodiafiltration, *HF* hemofiltration, *KDIGO* Kidney Disease: Improving Global Outcomes, *KFRT* kidney failure with replacement therapy, *KRT* kidney replacement therapy, *LDKT* living donor kidney transplant/transplantation, *MDRD* modification of diet in renal disease, *mGFR* measured GFR, *ND-CKD* non-dialysis CKD, *NDD-CKD* non-dialysis-dependent CKD, *PCR* protein-creatinine ratio, *PD* peritoneal dialysis, *PER* protein excretion rate, *pre-ESRD* pre-end-stage renal disease, *RF* renal failure, *RIFLE* risk injury failure loss of kidney function and end-stage kidney disease, *RRT* renal replacement therapy, *SCr* serum creatinine, TR tubular reabsorption, TS tubular secretion

^a^Journal style will dictate whether and when to abbreviate terms

^b^Ongoing discussion; may be revised by KDIGO AKI guideline update

^c^Ongoing discussion; may be revised by KDIGO AKD consensus conference

^d^Ongoing discussion; may be revised by KDIGO Glomerulonephritis guideline update

A guiding principle for the development of the glossary was patient-centeredness. The Health and Medicine Division of the US National Academies of Sciences defines patient-centered care as “providing care that is respectful of, and responsive to, individual patient preferences, needs and values, and ensuring that patient values guide all clinical decisions” [9]. One of the 10 general principles recommended for redesign of the health system is “Knowledge is shared and information flows freely. Patients should have unfettered access to their own medical information and to clinical knowledge. Clinicians and patients should communicate effectively and share information.” In principle, the terms used to describe kidney function and disease should be understandable to all, with acknowledgment of variation in the level of health literacy. Use of multiple terms with similar meaning can lead to confusion, as can use of terms that forecast the future (such as “pre-dialysis”) rather than describe the present. However, convergence of multiple names into an accepted set of terms does require that users of the glossary are willing to accept that labels that have been preeminent historically, and that may be more familiar or memorable even now, should now be superseded [10].

Of equal importance to patient-centeredness in the development of the glossary was precision, which can generally be defined as exactness or accuracy [10]. How medicine is defined and understood is changing rapidly from a descriptive, disease-based categorization in which multiple pathogenetic pathways may be conflated to a mechanism-based categorization that will promote more precise management of clinical problems. The latter approach, in which a molecular profile is added to the clinical and morphologic profile, has already revolutionized diagnosis and treatment in oncology. In nephrology, the ongoing Kidney Precision Medicine Project, funded by the National Institutes of Health, seeks to ethically obtain and evaluate kidney biopsies from participants with AKI or CKD; create a kidney tissue atlas; define disease subgroups; and identify cells, pathways, and targets for novel therapies [11]. As has occurred in oncology, it is anticipated that refinements that result in more precise disease descriptions will be incorporated into current nomenclature for kidney function and disease, rather than replace it altogether. Thus, although the glossary is designed to be consistent with current knowledge and stable enough to remain relevant for the foreseeable future, it is also intended to be sufficiently flexible to accommodate new vocabulary arising with advances in the field.

A central strength of the proposed glossary is that it is based on existing KDIGO definitions, classifications, and nomenclature for acute and chronic kidney disease. In addition, it was developed using the following: a systematic process, including articulation of a clear and transparent rationale (patient-centeredness and precision); capture of stakeholder viewpoints via patient focus groups [12] and a corresponding survey; a period of public comment on conference scope; and attainment of consensus among attendees at the conference. Although the recommendations are not likely to answer all concerns, the consensus among conference attendees was that standardizing scientific nomenclature is a necessary first step to improving communications among clinicians, researchers, and public health officials, and with patients, their families and caregivers, and the public.

Limitations of the proposed glossary are that it is restricted to English (nuances may be difficult to translate); only a limited number of stakeholders were able to participate, owing to practical reasons; it is not comprehensive (it does not include disease classification, dialysis, transplantation); and further specification is required for studies in children. For these and other reasons, we consider the current recommendations for a glossary to be an important starting point, and it will require future expansion and updating.

Achieving consensus among conference attendees, and publication of the conference report and glossary, is only the first step in implementation of a revised nomenclature. The glossary will be freely available on the KDIGO website (https://kdigo.org/conferences/nomenclature/; Table 2). Elements of the glossary will be included in online updates to the newly released (11th) edition of the *AMA Manual of Style* [13]. Medical journals adopting the recommendations will need to determine how to implement them, and this process will require education of editorial staff as well as proactive communication with authors, generally and with regard to specific manuscripts. If successful, further implementation in clinical practice, research, and public health will require more widespread dissemination and professional education. Improving communication with patients and the public will require efforts to improve patient education and health literacy for the public, and guide to communication with patients. Professional societies, industry, and patient advocacy organizations will be critical to these efforts.

Advances in research, particularly in precision medicine, will introduce a myriad of new terms and novel concepts requiring incorporation into disease definitions and classifications. In addition, the increasing prominence and participation of patient and caregiver communities in defining research and best practices in clinical care will further elucidate the characteristics of patient-centered terminology. Expanding and updating the KDIGO glossary can be accomplished as part of the activities of future KDIGO guideline workgroups and conferences.

## Electronic supplementary material

Below is the link to the electronic supplementary material.

Supplementary file1 (PDF 115 kb)
